# Personalized Treatment for Obstructive Sleep Apnea: Beyond CPAP

**DOI:** 10.3390/life14081007

**Published:** 2024-08-13

**Authors:** Margot Van Daele, Yannick Smolders, Dorine Van Loo, Charlotte Bultynck, Johan Verbraecken, Anneclaire Vroegop, Thérèse Lapperre, Sara Op de Beeck, Marijke Dieltjens, Olivier M. Vanderveken

**Affiliations:** 1 Department of Otorhinolaryngology, Head and Neck Surgery, Antwerp University Hospital, 2650 Edegem, Belgium; 2 Department of Translational Neurosciences, Faculty of Medicine and Health Sciences, University of Antwerp, 2000 Antwerp, Belgium; 3 Multidisciplinary Sleep Disorders Centre, Antwerp University Hospital, 2650 Edegem, Belgium; 4 Department of Respiratory Medicine, Antwerp University Hospital, 2650 Edegem, Belgium; 5 Laboratory of Experimental Medicine and Pediatrics, University of Antwerp, 2000 Antwerp, Belgium

**Keywords:** CPAP intolerance, obstructive sleep apnea, alternative treatment, personalized medicine

## Abstract

Obstructive sleep apnea (OSA) is a sleep disorder characterized by repetitive episodes of partial or complete obstruction of the upper airway during sleep. Continuous positive airway pressure (CPAP) is a method used as a first-line treatment for obstructive sleep apnea (OSA). However, intolerance and resistance to CPAP can limit its long-term effectiveness. Alternative treatments are available, such as Mandibular Advancement Devices (MADs), positional therapy, upper airway surgery, and maxillomandibular osteotomy. However, often less efficient in reducing the apnea-hypopnea index, the higher tolerance of and compliance to alternative treatment has resulted in the adequate treatment of OSA in CPAP-intolerant patients. This paper describes the protocol of a prospective single-center cohort study including adult patients with moderate to severe OSA (15 events/h ≤ apnea-hypopnea index (AHI) < 65 events/h) that failed to comply with CPAP therapy. Selected patients will be invited to the clinic to explore alternative treatment options where DISE will be a first step in further identifying upper airway collapse during sleep. By exploring alternative treatment options in CPAP-intolerant patients and systematically documenting their treatment paths, an algorithm can be defined to better guide patients towards personalized treatment for OSA. The follow-up is aimed at 5 years with an inclusion of 170 patients per year, including a drop-out rate of 15%. By leveraging a real-world database, this study aims to bridge the gap between research and clinical practice, facilitating the development of evidence-based guidelines and personalized treatment algorithms for CPAP-intolerant patients.

## 1. Introduction

Obstructive sleep apnea (OSA) is a sleep disorder characterized by repetitive episodes of partial or complete obstruction of the upper airway during sleep, leading to reduced or completely stopped airflow despite ongoing respiratory efforts. This results in disrupted sleep and intermittent hypoxia. This disorder is estimated to affect 17% of middle-aged women and 34% of middle-aged men [1]. Loud snoring, a precursor of OSA, is even more prevalent and is estimated to affect 40–60% of adult people [2]. OSA does not only cause symptoms such as excessive daytime sleepiness, loud snoring, and interrupted breathing during sleep. OSA can have significant health implications, including increased risk of hypertension, cardiovascular disease, and impaired cognitive function in the long term, as shown in several scientific studies [3,4,5]. Although 80% of patients remain undiagnosed, increasing awareness is seen over the last years [6].

OSA is diagnosed with an overnight polysomnography in a hospital setting or at home, in patients who are clinically and anamnestically suspected of OSA. Symptoms are habitual snoring, choking or gasping during sleep, excessive daytime sleepiness, inattention, and poor recall. Severity of OSA is measured using the apnea-hypopnea index (AHI), the number of apneas and hypopneas per hour of sleep. The diagnosis is confirmed if the AHI is at least five events per hour of sleep. Severity based on AHI is defined as follows: mild OSA (5 < AHI ≤ 15/h), moderate OSA (15 < AHI < 30/h) and severe OSA (AHI ≥ 30/h) [7].

Continuous positive airway pressure (CPAP) is a method used as first-line treatment for OSA. CPAP therapy significantly reduces disease severity, sleepiness, blood pressure, and sleepiness-related traffic accident occurrence, as well as improves the sleep-related quality of life in adults with sleep apnea [8].

However, intolerance and resistance to CPAP can limit its long-term effectiveness. Various factors contribute to CPAP intolerance, including mask discomfort, claustrophobia, nasal congestion, air leaks, pressure intolerance, and difficulty adjusting to the therapy [9]. Compliance failure is reported to be different in various countries. A recent nation-wide French database study showed that almost half of patients on CPAP therapy terminate the CPAP treatment after three years [10]. Therefore, alternative treatment options in CPAP-intolerant or non-compliant patients have gain increased interest and importance in daily practice, leading us towards a more personalized way of treatment.

Alternative treatments for OSA include Mandibular Advancement Devices (MADs), positional therapy, upper airway surgery, and maxillomandibular osteotomy [11,12,13,14]. MAD therapy has emerged as a second-line conservative non-invasive treatment method for the management of mild to moderate OSA, and lately even for severe OSA patients. MAD therapy consists of a titratable duo-block with separate upper and lower parts that are dynamically interconnected. This allows for a gradual protrusion of the lower jaw until the optimal position is reached. As such, patients can optimize the ideal position that allows symptoms (such as snoring and tiredness) to disappear and to tolerate protrusion as well. MAD therapy functions on increasing the upper airway volume, mainly the widening of the lateral walls of the velopharynx [15]. 

Positional therapy was known in the past as placing bulky masses on the back of patients, also known as the “tennis ball technique” to prevent supine sleep [16]. A new form of positional therapy is the sleep position trainer (SPT) and can be indicated for patients with positional OSA, defined as a supine AHI that is at least twice as high as compared to the AHI in other positions. SPT is a small, lightweight device that the patient can wear around the chest or neck using a strap during the night. A three-dimensional digital accelerometer is used to measure the sleep position of the patient. When the patient is laying on the back, the device responds with a vibration stimulus to initiate body movement. The device stimulates with a gradually increasing strength and stimulus duration, until a non-supine position is detected. The aim is to make the patient switch positions without full awareness and without waking the patient [16,17]. SPT was found to effectively diminish the percentage of supine sleep, reduce the overall AHI, and improve subjective sleepiness in patients with position-dependent OSA [18]. 

Palatal surgery is a surgical treatment option for OSA patients that has known different techniques over the last couple of years. Barbed reposition pharyngoplasty (BRP) was developed in 2015 and shows promising results in well-selected patients. It is a simple and secure procedure that can be used as a standalone therapy or in combination with another therapy such as MAD. In brief, the method entails that the posterior pillars are displaced in a more lateral and anterior position to enlarge the oropharyngeal inlet as well as the retropalatal space. Then, the posterior pillar is suspended to the pterygomandibular raphe [19,20]. Expansion sphincter pharyngoplasty (ESP) is almost as efficient as BRP, but it comes with a higher risk of complications [20]. The results of ESP are better when combined with other techniques. The procedure always starts with a bilateral tonsillectomy, followed by identifying and isolating the palatopharyngeal muscle and creating a palatopharyngeal muscle rotation flap [20,21]. 

Surgical treatment of the upper airway is common for OSA. Hypoglossal nerve stimulation (HNS) or upper airway stimulation is an innovative therapy for patients with moderate to severe OSA. A surgically implanted device will generate respiration synchronized electrical pulses through a generator that is implanted in the upper right chest. These electrical pulses are sent to the hypoglossal nerve to stimulate tongue protrusion and widening of the pharyngeal wall to alleviate upper airway collapse [22,23]. The device consists of a pulse generator, which is implanted just below the right clavicula, and two leads. The sensing lead will be implanted in the second intercostal space close to the right lung to detect breathing. The other lead contains a cuff which will be placed around the protruding branches of the hypoglossal nerve to guide pulses through the nerve and to generate muscle contraction [24]. Postoperatively, the device can be activated at night with a remote after one month. Variable electrode configurations can be installed to obtain an optimal result. The device will be set up during a sleep study one month to six weeks after the procedure. The first ideas about the effect of the HNS can then be observed and the configurations can be changed. 

Patients are suitable for HNS following these exclusion criteria: a complete concentric collapse of the palate during DISE, an AHI ≥ 65/h, a BMI ≥ 32 kg/m^2^, and not having tried CPAP and MAD therapy in the past. Also, confounding sleep disorders and ≥15% central apneas need to be excluded.

Combination therapy for the treatment of obstructive sleep apnea (OSA) is occasionally necessary to achieve optimal outcomes, although it applies to a relatively small subset of patients. For some individuals, a single treatment modality may not sufficiently alleviate symptoms or address the multifactorial nature of their airway obstruction. In such cases, a tailored approach that integrates multiple therapies can be beneficial. For example, combining hypoglossal nerve stimulation, which targets neuromuscular control of the upper airway, with sleep position therapy, which mitigates positional airway collapse, can provide synergistic effects [25]. This dual strategy may be particularly effective for patients with complex anatomical or physiological contributors to their OSA, offering improved symptom relief and enhanced adherence compared to monotherapy. By recognizing and addressing the unique needs of this patient group, healthcare providers can deliver more comprehensive and effective treatment plans [26]. 

In a case where any alternative treatment to CPAP is considered, drug-induced sleep endoscopy (DISE) is often performed. DISE is a clinical standard diagnostic procedure to study the collapse pattern of the upper airway in patients with snoring and/or sleep apnea problems during drug-induced sleep. It allows us to visualize the upper airway using sedative drugs, which mimic natural sleep [27]. DISE allows us to identify an OSA patient and the characteristics of the upper airway, as collapse patterns differ from patient to patient. With information obtained from DISE, targeted therapy can be considered which is preferred in current days where personalized medicine is the aim [28]. 

The primary objective of this study is to establish a real-world database that collects patient data seamlessly within the framework of standard clinical care. By integrating data collection into routine patient management, we aim to capture valuable insights into the effectiveness and patient-reported outcomes of alternative treatments for CPAP intolerance. 

By this approach, we seek to bridge the gap between research and clinical practice, facilitating the development of evidence-based guidelines and personalized treatment algorithms for patients with CPAP intolerance. By leveraging real-world data, we can improve the quality of care and ultimately enhance the well-being of individuals affected by OSA.

## 2. Materials and Methods

### 2.1. Study Design 

This study is an observational, single-center cohort study including patients with moderate to severe OSA (AHI ≥ 15 events/h and < 65 events/h) that have failed to comply with CPAP therapy. The study can be defined as a real-world database that will collect long-term data of patients following routine clinical practice. To the best of our knowledge, this will be the first study to register data on CPAP-intolerant patients and their treatment path in a long-term follow-up. 

### 2.2. Participants

**Figure 1:**
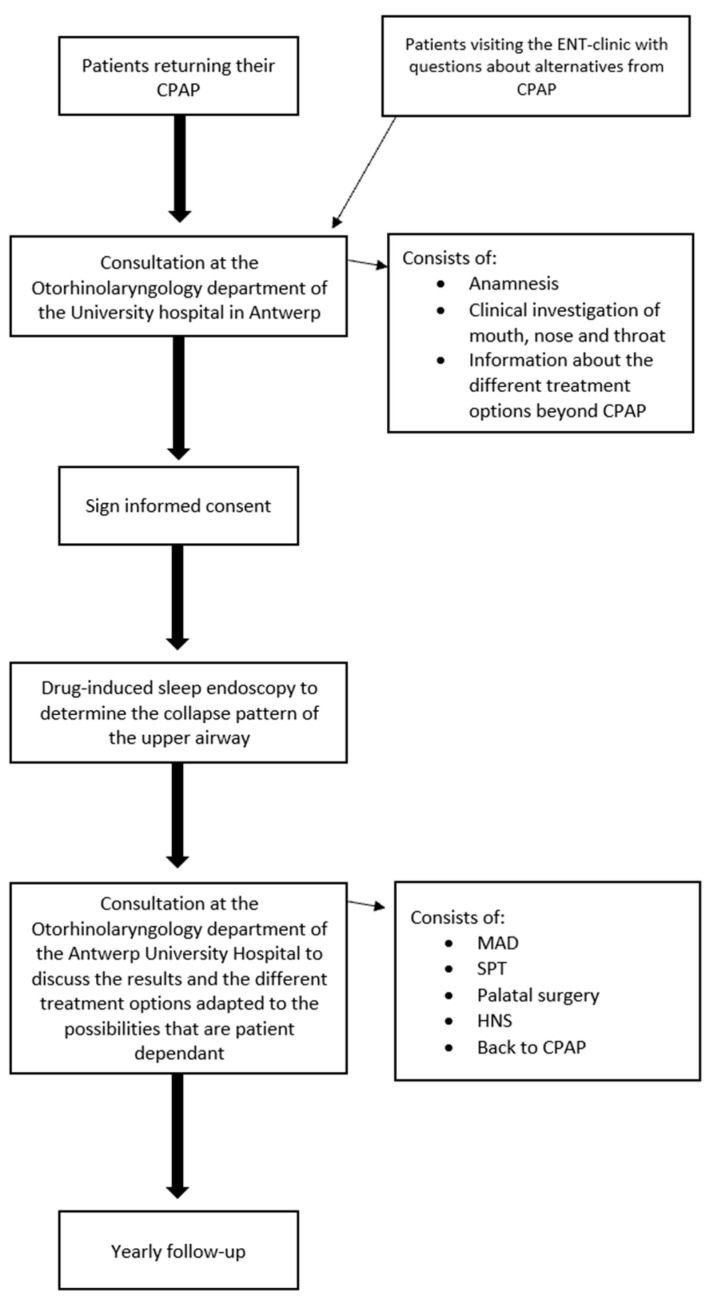
Clinical pathway during the study.

Inclusion criteria are adult patients (age starting from 18 years old) with moderate to severe OSA (AHI ≥ 15/h) who have been shown to be CPAP-intolerant or non-compliant and are willing to explore alternative treatment options. When returning the CPAP device, patients will be informed about the possibility of a consultation regarding alternative treatment options (Figure 1). Patients will be contacted by phone to ask for the main reason to cease CPAP therapy and, if interested in alternative therapy, will be invited to a designated consultation at the ENT department. Factors for CPAP cessation will be documented and analyzed in further investigation to better predict CPAP failure and adherence to alternative treatment. All patients will be asked to provide written informed consent. 

Exclusion criteria will be patients who are still on CPAP therapy, pediatric patients, newly diagnosed patients who have not had CPAP before, and patients with AHI ≥ 65 events/h.

### 2.3. Intervention

No intervention will be performed apart from the normal clinical pathway for OSA patients. The institutional ethics committee has approved the study protocol and written informed consent is obtained from all participants. 

One of the treatment options is MAD therapy. In our clinic, home polygraphy is performed after 3 months to objectively evaluate the effect of the treatment. 

If MADs had failed in the past or is not considered an option, surgical options (such as BRP, ESP, or HNS therapy) can be considered depending on the collapse pattern of the upper airway, as seen during DISE. HNS therapy has strict reimbursement criteria and is only indicated for patients with moderate to severe OSA (AHI 15–65 events per hour), who do not tolerate CPAP or MAD. Contraindications are patients with central sleep apnea, sleep-related hypoxia or hypoventilation, a body mass index above 32 kg/m^2^, and patients with a complete concentric collapse pattern of the palate [12]. For patients with supine-dependent OSA, treatment with a SPT can be very effective [17]. 

### 2.4. Diagnostic Work-Up

#### 2.4.1. Polysomnography (PSG) 

The standard method to objectively diagnose sleep disorders, including OSA, is a full night attended sleep study or type I PSG. During a PSG, several parameters are measured including electroencephalogram, electro-oculogram, electromyogram, electrocardiogram, and pulse oximetry, as well as airflow/respiratory effort and body position. The severity of OSA is expressed by the AHI which counts the number of apneas and hypopneas per hour of sleep. During a PSG, a distinction is made between obstructive and central apneas [29]. Every PSG is scored manually according to the ASSM 2012 rules [30]. Polysomnography also documents other sleep disorders, which will be registered in our protocol.

#### 2.4.2. Polygraphy (PG) 

A polygraphy or home sleep study is a sleep study that is performed in the patients’ home. It is an alternative and accepted method for diagnosing OSA in adults [31]. We know that measurement errors are possible with PG compared to PSG because there is no analysis of the standard sleep stages. During PG, the recording time rather than sleep time is used. Knowing this, a PG can underestimate the OSA [32]. 

#### 2.4.3. Drug-Induced Sleep Endoscopy 

Patients will undergo a DISE to evaluate the collapse pattern of the upper airway. For example, to be suitable for HNS and MAD, a complete concentric collapse at the level of the palate needs to be ruled out. The use of a simulation bite during DISE can simulate the effect of potential treatment with a MAD. In our clinic, dental impressions are made as titratable bites that simulate the effect of an MAD. During DISE, the starting position of the simulation bite is at ‘maximal comfortable protrusion’ (MCP), which is the protrusion that is defined by the patient as a comfortable position for the lower jaw. From this position, the simulation bite is titrated forward to reproduce the effect of a MAD [33]. 

After DISE, targeted therapy will be considered with the patient. Follow-up for the chosen treatment will be as in standard clinical practice. The clinical pathway for MADs and HNS is described by, respectively, Ten Berge [34] and Vanderveken et al. [35]. The targeted therapy chosen after consultation will be registered and followed in the database to obtain an overview of the treatments in the CPAP-intolerant patients. 

### 2.5. Questionnaires

#### 2.5.1. Epworth Sleepiness Scale (ESS) 

The ESS is a self-administered questionnaire with 8 questions. Respondents are asked to rate, on a 4-point scale (0–3), the likelihood of dozing off or falling asleep while engaging in eight different activities. The ESS score (the sum of 8 item scores, 0–3) can range from 0 to 24. The higher the ESS score, the higher that person’s average sleep propensity in daily life, or their ‘daytime sleepiness’ [36]. A score > 10 may indicate the presence of sleep disorders and a score > 16 is associated with severe symptoms, related to OSA, narcolepsy, or idiopathic hypersomnia [37]. 

#### 2.5.2. Visual Analogue Scale (VAS) 

The VAS on snoring is a Likert scale ranging from 0 (no snoring) to 10 (socially disturbing snoring), and it is used to score subjective symptoms [38,39].

#### 2.5.3. Functional Outcome of Sleep Questionnaire (FOSQ) 

The FOSQ-30 is a quality-of-life questionnaire to determine functional status in adults. Measures are designed to assess the impact of disorders of excessive sleepiness on multiple daily activities and the extent to which these are improved by effective treatment. Several categories are questioned such as activity level, vigilance, intimacy and sexual relationships, general productivity, social outcome. Each item has a 1 (extremely difficult) to 4 point (no difficulty) scale, where the total score equals the mean scores for each subscale multiplied by the number of subscales answered. Scoring < 18 is considered abnormal, and a lower score implies more effect of sleepiness on daily life [40,41]. 

Patients will be asked at the first consultation for the arguments to cease CPAP treatment and how long the CPAP treatment lasted. In addition, data will be collected from polysomnography (PSG), the Epworth Sleepiness Scale (ESS), Visual Analogue Scale (VAS) for snoring and Functional Outcome of Sleep Questionnaire (FOSQ) questionnaires. These questionnaires will be filled in during the first consultation and repeated after established treatment, dependent of the clinical pathway of the given therapy. From the start of adequate treatment, patients will have a yearly follow-up in clinic, or sooner in the case of problems.

#### 2.5.4. Statistical Analysis 

The primary aim is to offer alternative treatment to CPAP-intolerant patients and to gain more insight in why and how patients tolerate alternative treatment. The secondary aim is to identify which patients are likely to be successful for certain treatments, depending on their personal and OSA characteristics. Descriptive statistics will be used to determine patient characteristics and normality. Univariate and linear regression analysis will be used to assess therapy outcome and significance of treatment effect. Analyses will be adjusted for known covariates. 

Analysis of the data will be carried out through the JMP software (Statistical Discovery LLC., Cary, NC, USA). 

Based on data of the Multidisciplinary Sleep Disorders Centre, around 1000 patients start CPAP therapy every year. Taking into account that an expected 20% of these patients will quit CPAP therapy, we aim to include 200 patients a year. Taking into account a targeted sample size of 1000 patients and a drop-out rate of 15%, inclusion will continue for 6 years. 

## 3. Results

This study protocol represents a pioneering effort to address the ongoing need for comprehensive data on CPAP intolerance and alternative treatment options in OSA patients. By leveraging this database, we can optimize patient care and improve the management of OSA, leading to better health outcomes and quality of life for affected individuals. As far as our knowledge, this is the first broad study to register CPAP-intolerant patients and their clinical treatment path afterwards. 

### Protocol

CPAP-intolerant patients who hand in their CPAP device at the Department of Pneumology will be referred to the ENT Department. During this conversation they will be asked about their motivation to stop CPAP and whether they have interest in alternative treatment options. Interested patients will be planned for consultation to discuss and explore alternatives. 

DISE will be conducted to evaluate the collapse pattern of the upper airway. This diagnostic procedure will provide insights into the collapse pattern during sleep. The results of this procedure will be used to guide the patient towards different treatment options. 

Depending on the DISE outcome, the possible treatment options will be discussed with the patient. These options may include but are not limited to mandibular advancement device therapy, positional therapy, upper airway surgery, or other interventions tailored to the individual patients’ needs. A possible option, in case no other suitable treatment is feasible, can be CPAP (or eventually Bilevel CPAP) again as well as combination therapy if monotherapy does not suffice. For example, in supine-dependent hypoglossal nerve stimulation patients, positional therapy will be added to optimize treatment success.

Subsequent follow-up appointments will be scheduled to evaluate the patients’ compliance and subjective results with the chosen therapy. These regular assessments will allow for adjustments in treatment as necessary, aiming to optimize long-term outcomes and patient comfort.

For MAD therapy, this is assessed in our clinic through a 3-month polygraphy, such as for positional therapy. For HNS, there would be a 3-month polysomnography in clinic, followed by a yearly polysomnography. 

## 4. Discussion

This study aims to establish a comprehensive real-world database capturing long-term data on patients with moderate to severe obstructive sleep apnea (OSA) who are intolerant or non-compliant with continuous positive airway pressure (CPAP) therapy. By integrating data collection into routine clinical practice, we seek to evaluate the effectiveness and patient-reported outcomes of alternative treatments, ultimately aiming to enhance personalized treatment approaches for OSA. 

The high prevalence of OSA and its significant health implications underscore the importance of effective management strategies. Although CPAP therapy remains the standard for OSA treatment due to its efficacy in reducing disease severity and improving quality of life, intolerance and non-compliance significantly limit its long-term effectiveness. The reasons for CPAP intolerance can be multifactorial and may include mask discomfort, claustrophobia, nasal congestion, air leak, pressure intolerance, and difficulty adjusting to the therapy [9]. Although, certainly in severe OSA, alternative treatment does not always seem to be able to reach the same results as CPAP therapy, a higher compliance rate can sometimes offer a treatment that is as efficient as CPAP [42]. 

Given these limitations, alternative treatment options are gaining traction. Mandibular advancement devices (MADs), positional therapy, upper airway surgery, and hypoglossal nerve stimulation (HNS) have emerged as viable alternatives. MADs, for instance, have shown promise in increasing upper airway volume and reducing OSA symptoms. Positional therapy, particularly using sleep position trainers (SPTs), effectively reduce supine-dependent OSA by promoting non-supine sleep positions through gentle vibrational feedback. Upper airway surgery techniques, such as barbed reposition pharyngoplasty (BRP) and expansion sphincter pharyngoplasty (ESP), offer structural interventions to prevent airway collapse during sleep. HNS, an innovative therapy involving a surgically implanted device, stimulates the hypoglossal nerve to maintain airway patency during sleep [12,13,14,15]. 

In the intention of personalizing sleep medicine and offering patients alternatives, it is crucial to recognize that for some individuals, CPAP remains the only viable treatment. CPAP’s role as the standard in OSA management is reinforced by its unparalleled effectiveness in several scenarios. Studies consistently demonstrate that CPAP significantly reduces the apnea-hypopnea index (AHI) and improves oxygen saturation levels in patients with severe OSA, more effectively than alternative treatments like oral appliances or positional therapy [43,44]. For these patients, the potential health risks associated with insufficiently treated severe OSA, such as cardiovascular complications and increased mortality, make CPAP the only reliable option. Certainly, when patients are characterized by a mixed complex sleep apnea syndrome, where both central and obstructive apneas are seen, only CPAP will guarantee treatment of both components [44]. 

Furthermore, when alternative treatments fail, CPAP remains the fallback option. Oral appliances might not provide sufficient relief for patients with severe OSA or specific anatomical challenges and multilevel collapse during DISE. Surgical interventions, while beneficial for some, do not guarantee success for all patients [44]. 

In conclusion, while exploring alternatives to CPAP for OSA patients is valuable, it is essential to keep in mind that, in some cases, this study will include patients that will be referred for CPAP therapy if, after careful evaluation, this is thought to be the best option. Its effectiveness in reducing AHI, improving oxygen saturation, and managing comorbid conditions solidifies CPAP’s role as a critical component in the management of complex and mixed OSA [44]. 

Drug-induced sleep endoscopy (DISE) plays a crucial role in personalizing OSA treatment by visualizing upper airway collapse patterns and guiding the selection of appropriate therapies. This step is essential in further evaluation and exploring new treatment options for OSA patients. In complex OSA patients, DISE is indispensable in pinpointing the exact location(s) of collapsibility in the upper airway. As such, other treatment can be completely explored and ruled out before referring patients back to CPAP therapy. For example, when a patient is dentally unsuitable for MAD treatment, and there is a complete concentric collapse (CCC) at the level of the palate, two alternative treatment options are ruled out. A possible solution for this problem is to perform palatal surgery to alleviate the CCC and to become a good candidate for hypoglossal nerve stimulation [45,46]. In case the patients do not tend to undergo surgery, they will be referred for CPAP therapy. 

From the literature, it is known that drug-induced sleep endoscopy (DISE) has its clinical limitations. One significant drawback is that DISE does not measure REM sleep, a critical phase of sleep during which muscle tone is further reduced and airway collapsibility may increase. This omission means that DISE might not fully replicate the natural sleep conditions where the collapse of the upper airway might be more pronounced. Additionally, while natural sleep endoscopy (NSE) would provide the most accurate representation of airway behavior during sleep, it is not feasible in daily clinical practice due to the inability to control sleep stages and patient movement during a natural sleep state. However, as seen in the literature, NSE can show altering results in collapse patterns compared to DISE. Despite these limitations, DISE remains the best option for clinicians to visualize and assess the upper airway dynamically, allowing for targeted and personalized treatment interventions for OSA patients [47]. 

By leveraging a real-world database, this study aims to bridge the gap between research and clinical practice, facilitating the development of evidence-based guidelines and personalized treatment algorithms for CPAP-intolerant patients. The comprehensive data collected will inform future treatment strategies, improve patient outcomes, and contribute to the growing body of knowledge on OSA management. 

It is important to acknowledge some limitations of this study protocol. Firstly, the observational nature of the study may introduce inherent biases and confounding factors that could affect the interpretation of results. Secondly, the generalizability of findings may be limited by the heterogeneous nature of OSA patients and variations in clinical practices. Despite these limitations, we believe that this study has the potential to significantly contribute to our understanding of CPAP failure and alternative treatment strategies in OSA patients.

## 5. Conclusions

This study will evaluate the broader field of possibilities beyond CPAP in patients with moderate to severe OSA. Given the prospective nature of data in the study, we will be able to fully characterize these patients and identify important and potentially new predictive factors for treatment outcomes with several therapies for OSA. The advantages of each of the individual pre-treatment investigations will be combined with the aim of translating it into an optimal selection procedure, leading to an evidence-based decision making and targeted treatment of patients with OSA.

## Data Availability

No new data were created or analyzed in this study. Data sharing is not applicable to this article.
